# Influence of cardiovascular risk burden on pulmonary function trajectory: role of physical and social activities

**DOI:** 10.18632/aging.204201

**Published:** 2022-08-04

**Authors:** Yang Bai, Jiao Wang, Ruixue Song, Zhangyu Wang, Xiuying Qi, Aron S. Buchman, David A. Bennett, Weili Xu

**Affiliations:** 1 Department of Epidemiology and Biostatistics, School of Public Health, Tianjin Medical University, Tianjin 300070, China; 2 Tianjin Key Laboratory of Environment, Nutrition and Public Health, Tianjin 300070, China; 3 Center for International Collaborative Research on Environment, Nutrition, And Public Health, Tianjin 300070, China; 4 Shandong Provincial Clinical Research Center for Emergency and Critical Care Medicine, Institute of Emergency and Critical Care Medicine of Shandong University, Qilu Hospital of Shandong University, Jinan 250012, Shandong, China; 5 Rush Alzheimer’s Disease Center, Chicago, IL 60612, USA; 6 Department of Neurobiology, Care Sciences and Society, Karolinska Institutet, Stockholm 17177, Sweden

**Keywords:** Framingham general cardiovascular risk score, pulmonary function, physical activity, social activity, community-based cohort study

## Abstract

The impact of cardiovascular risk burden on long-term trajectories of pulmonary function (PF) remains unclear. We examined the association of cardiovascular risk burden assessed by Framingham general cardiovascular risk score (FGCRS) with PF decline and explored whether cardiovascular diseases (CVD), physical and social activities play a role in the association. Within the Rush Memory and Aging Project, 1,442 participants (mean age:79.83) were followed up to 22 years. FGCRS at baseline was calculated and categorized into tertiles. Composite PF was measured annually based on peak expiratory flow, forced expiratory volume in one second, and forced vital capacity. We found that the highest FGCRS was associated with faster PF decline (β: -0.013, 95% CI: -0.023 to -0.003) compared with the lowest FGCRS. There were significant interactions between higher FGCRS and low level of physical/social activity (β: -0.014, 95% CI: -0.026 to -0.003)/(β: -0.020, 95% CI:-0.031 to -0.009) or CVD(β: -0.023, 95% CI:-0.034 to -0.011) compared to the low FGCRS with high level of physical/social activity or without CVD (P-interaction<0.05). Our results suggest that higher cardiovascular risk burden is associated with a faster PF decline, especially among people with CVD. High level of physical activity and social activity appears to mitigate this association.

## INTRODUCTION

Aging is inevitably attended by a decline in pulmonary function (PF), owing to various factors, including lung elasticity damage and respiratory muscle weakness [1]. To evaluate the respiratory system function, PF is comprehensively measured by forced expiratory volume in one second (FEV1), forced vital capacity (FVC), and peak expiratory flow (PEF) [2]. Furthermore, low PF has been linked to decreased general health and quality of life and increased all-cause mortality in older adults [3–5].

Growing evidence suggests that traditional cardiovascular risk factors, including smoking, hypertension, dyslipidemia, and diabetes mellitus, may be linked to poor FEV1, FVC, or PEF, respectively [6–9], though with inconsistent results [10, 11]. Cardiovascular risk factors are well known to be interrelated, and therefore, a comprehensive indicator makes it possible to evaluate the overall cardiovascular burden. The Framingham general cardiovascular risk score (FGCRS), combining age and sex with traditional cardiovascular risk factors, is a prediction scoring algorithm used to thoroughly assess the burden of cardiovascular risk and the likelihood of developing cardiovascular disease (CVD) [12]. However, there are still unanswered questions regarding whether and to what extent FGCRS may affect the long-term trajectories of PF.

Although the incidence of cardiovascular disease in older adults is increasing, modifiers of its association with PF have not been well studied. Previous studies have shown that poorer physical activity [13, 14] and social activity [15] are related to poorer PF among older adults. Consequently, it is essential to further explore whether physical and social activities have a moderating effect on the association of FGCRS with PF decline.

In the current study, using data from the Memory and Aging Project (MAP), we 1) examine the relationship of FGCRS-assessed cardiovascular risk burden with long-term trajectories of PF, and 2) explore the role of CVD, physical and social activities in the association between FGCRS and PF.

## RESULTS

### Baseline characteristics

Among the 1,442 participants with a mean age of 79.83±7.47 years, the range of FGCRS was 4-28 at baseline. Participants with the highest FGCRS were older, had higher proportions of males and smokers, and had lower educational attainment, physical activity, social activity, and HDL-C, compared to those with the lowest FGCRS. Furthermore, those with the highest FGCRS also tended to have higher BMI and SBP, and have hypertension, diabetes, stroke, and heart diseases (Table 1).

**Table 1:** Characteristics of the study population by tertiles of the Framingham general cardiovascular risk score (FGCRS) at baseline (N =1,442).

**Characteristics**	FGCRS*			***P*-value**
	**Lowest (N=535)**	**Middle (N=442)**	**Highest (N=465)**	
Age, yrs	77.67 ± 8.52	80.99 ± 6.74	81.20 ± 6.19	<0.001
Female	474 (88.60)	327 (73.98)	275 (59.14)	<0.001
Education, yrs	15.11 ± 2.99	14.75 ± 3.23	14.66 ± 3.22	0.025
BMI, kg/m^2^	26.75 ± 5.25	27.39 ± 5.44	28.13 ± 4.98	<0.001
Alcohol consumption, g	1.08 (0.00, 6.04)	0.00 (0.00, 5.83)	0.00 (0.00, 5.18)	0.096
Smoking status				0.033
Never	308 (57.57)	274 (61.99)	276 (59.35)	
Former smoker	220 (41.12)	160 (36.20)	171 (36.77)	
Current smoker	7 (1.31)	8 (1.81)	18 (3.87)	
SBP, mm Hg	123.14 ± 12.59	134.52 ± 14.28	147.20 ± 16.36	<0.001
HDL-C, mg/dl	65.37 ± 16.72	60.99 ± 18.22	53.44 ± 18.01	<0.001
TC, mg/dl	189.57 ± 34.73	193.95 ± 43.78	191.85 ± 46.62	0.576
Pulmonary function	-0.02 (-0.61, 0.52)	-0.12 (-0.63, 0.56)	0.05 (-0.54, 0.69)	0.132
FVC	0.09(-0.53,0.79)	0.01(-0.54,0.72)	0.19(-0.48,0.98)	0.141
FEV1	0.08(-0.48,0.80)	0.04(-0.52,0.78)	0.19(-0.44,0.99)	0.196
PEF	0.03(-0.59,0.60)	-0.01(-0.60,0.62)	0.03(-0.59,0.60)	0.452
Hypertension	242 (45.23)	306 (69.23)	401 (86.24)	<0.001
Diabetes	19 (3.55)	39 (8.82)	146 (31.40)	<0.001
Stroke	32 (6.71)	34 (8.29)	54 (12.19)	0.012
Congestive heart failure	20 (3.85)	26 (6.24)	17 (3.97)	0.166
Heart diseases	29 (5.42)	43 (9.73)	60 (12.93)	<0.001
Depression	108 (20.19)	77 (17.42)	75 (16.13)	0.231
Physical activity, h/week	2.92 (1.04, 5.17)	2.75 (1.00, 4.67)	2.33 (0.75, 4.33)	0.037
Social activity	2.80 (2.33, 3.00)	2.67 (2.20, 3.00)	2.50 (2.17, 3.00)	<0.001

Values are mean ± SD, n (%), or median (interquartile range).

*FGCRS categories: lowest group (4 to 13); middle group (14 to 16); highest group (17 to 28).

Abbreviations: BMI, Body mass index; HDL-C, High-density lipoprotein cholesterol; SBP, Systolic blood pressure; TC, Total cholesterol; FVC, Forced vital capacity; FEV1, Forced expiratory volume in one second; PEF, Peak expiratory flow.

Missing data: BMI = 25; Stroke = 112; Congestive heart failure = 77; Heart diseases = 1.

### Association of FGCRS with PF

During a median follow-up of 7 years, when FGCRS was treated as a continuous variable, higher FGCRS was associated with a faster decline in PF (β: -0.002, 95% CI: -0.003 to -0.000) and a faster decline in FVC (β: -0.001, 95% CI: -0.002 to -0.000) and FEV1 (β: -0.001, 95% CI: -0.002 to -0.000) over time.

**Figure 1:**
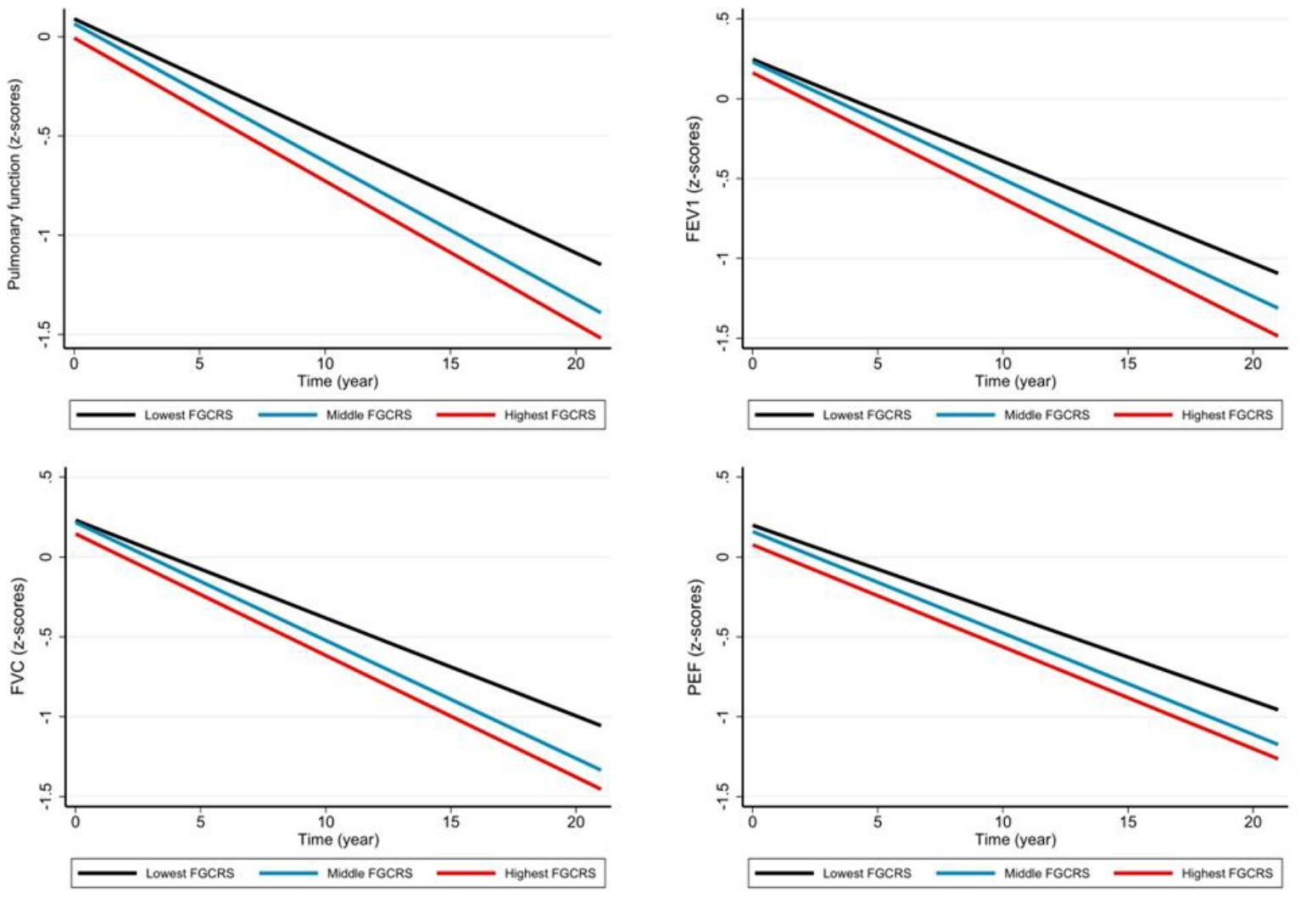
**Pulmonary function trajectories and different domains by Framingham general cardiovascular risk score (FGCRS) tertiled.** Note: Trajectories represent β-coefficients from linear mixed-effect models adjusted for sex, age, education, body mass index, alcohol consumption, physical activity, social activity, depression, stroke, congestive heart failure, and heart disease, with the lowest FGCRS group as reference group. Abbreviations: FVC, Forced vital capacity; FEVl, Forced expiratory volume in one second; PEF, Peak expiratory flow.

Participants with middle/highest FGCRS experienced an accelerated decline in PF (β: -0.010, 95% CI: -0.020 to -0.001)/ (β: -0.013, 95% CI: -0.023 to -0.003) and FVC (β: -0.008, 95% CI: -0.014 to -0.001)/ (β: -0.009, 95% CI: -0.016 to -0.002), compared to those with lowest FGCRS. Moreover, participants with highest FGCRS had a faster decline in FEV1 (β: -0.008, 95% CI: -0.014 to -0.002), compared to those with lowest FGCRS. The association between high FGCRS and PEF was not significant (Table 2 and Figure 1). The results of covariates were expressed in Supplementary Table 3.

**Table 2:** Association of the Framingham general cardiovascular risk score (FGCRS) with the changes of pulmonary function.

**FGCRS**	**Pulmonary function**	**FEV1**	**FVC**	**PEF**
	β (95% CI)*	β (95% CI)*	β (95% CI)*	β (95% CI)*
Baseline				
Continuous FGCRS	-0.017^†^ (-0.027 to -0.006)	-0.009^†^ (-0.016 to -0.003)	-0.010^†^ (-0.017 to -0.002)	-0.018^†^ (-0.030 to -0.006)
Categories FGCRS				
Lowest	Reference	Reference	Reference	Reference
Middle	-0.026 (-0.117 to 0.065)	-0.010 (-0.067 to 0.047)	-0.010 (-0.073 to 0.054)	-0.041 (-0.144 to 0.061)
Highest	-0.094^†^ (-0.188 to -0.001)	-0.045 (-0.104 to 0.014)	-0.050 (-0.115 to 0.015)	-0.120^†^ (-0.226 to -0.015)
Longitudinal				
Continuous FGCRS × time	-0.002^†^ (-0.003 to -0.000)	-0.001^†^ (-0.002 to -0.000)	-0.001^†^ (-0.002 to -0.000)	-0.001 (-0.002 to 0.000)
Categories FGCRS × time				
Lowest	Reference	Reference	Reference	Reference
Middle	-0.010^†^ (-0.020 to -0.001)	-0.005 (-0.011 to 0.001)	-0.008^†^ (-0.014 to -0.001)	-0.008 (-0.021 to 0.004)
Highest	-0.013^†^ (-0.023 to -0.003)	-0.008^†^ (-0.014 to -0.002)	-0.009^†^ (-0.016 to -0.002)	-0.009 (-0.021 to 0.004)

*Model adjusted for sex, age, education, body mass index, alcohol consumption, physical activity, social activity, depression, stroke, congestive heart failure, and heart diseases.

^†^*P* < 0.05.

Abbreviations: FVC, Forced vital capacity; FEV1, Forced expiratory volume in one second; PEF, Peak expiratory flow.

### Role of CVD, physical activity, and social activity in the FGCRS-PF association

As middle and highest FGCRS were both related to PF decline, they were combined into one group as high FGCRS in the joint effect analyses. In joint effect analyses (Table 3), there were significant interactions between higher FGCRS and low level of physical/social activity (β: -0.014, 95% CI: -0.026 to -0.003, *P*-interaction= 0.048)/ (β: -0.020, 95% CI: -0.031 to -0.009, *P*-interaction= 0.016) or CVD (β: -0.023, 95% CI: -0.034 to -0.011, *P*-interaction= 0.017) compared to the low FGCRS with high level of physical/social activity or without CVD. However, there was no interaction between FGCRS and smoking on PF (*P* > 0.05) (Supplementary Table 4).

**Table 3:** Joint effects of high Framingham general cardiovascular risk score (FGCRS) with cardiovascular disease (CVD), physical activity and social activity in relation to the decline on pulmonary function.

**Joint exposure**		**No. of subjects**	Pulmonary function β (95% CI)*
**FGCRS**	**Physical activity**		
Low	High	279	Reference
	Low	256	-0.005 (-0.018 to 0.008)
High	High	443	-0.010 (-0.021 to 0.001)
	Low	464	-0.014^†^ (-0.026 to -0.003)
*P*-interaction= 0.048			
**FGCRS**	**Social activity**		
Low	High	269	Reference
	Low	266	-0.006 (-0.018 to 0.007)
High	High	377	-0.006 (-0.017 to 0.005)
	Low	530	-0.020^†^ (-0.031 to -0.009)
*P*-interaction= 0.016			
**FGCRS**	**CVD**		
Low	No	462	Reference
	Yes	73	0.002 (-0.017 to 0.020)
High	No	710	-0.008^†^ (-0.016 to -0.000)
	Yes	197	-0.023^†^ (-0.034 to -0.011)
*P*-interaction= 0.017			

*Model adjusted for sex, age, education, body mass index, alcohol consumption, depression, as well as physical activity, social activity, and CVD, if applicable.

^†^*P* < 0.05.

### Sensitivity analysis

The results were not much altered when we repeated the analyses by excluding PF observations at baseline and within the first 2 years during the follow-up (Supplementary Table 5).

## DISCUSSION

In the long-term population-based longitudinal cohort study among the elderly, we found that 1) higher FGCRS-assessed cardiovascular risk burden was related to faster decline of composite PF, FVC, and FEV1, especially among people with CVD, and 2) there were significant interactions between FGCRS and physical/ social activity, and high levels of physical and social activities could mitigate PF decline related to higher FGCRS. Our findings reveal the adverse effect of vascular risk burden on pulmonary health and highlight the importance of active life in preventing pulmonary dysfunction among older people with a higher vascular risk burden.

Previous studies suggest that single cardiovascular risk factors (e.g., smoking, hypertension, diabetes, and dyslipidemia) are related to decreased lung function, with some inconsistent results [6–9]. A cohort study suggested that smoking is not associated with FVC decline [10]. In addition, some longitudinal studies indicated that hypertension, diabetes, and dyslipidemia are not associated with FEV1, FVC, or PEF decline [11, 16]. In these studies, PF was assessed using a single PF index without considering the use of a comprehensive combination of FEV1, FVC, and PEF to reflect the function of some respiratory muscles [17]. Given that cardiovascular risk factors often interact with each other, in the present study, FGCRS, as a comprehensive indicator, was used to assess multiple cardiovascular risk factors. However, the relationship between comprehensive cardiovascular risk indicators and PF decline has not yet been studied. In the present study, we found that higher FGCRS-assessed cardiovascular risk burden was related to faster PF decline (including FEV1 and FVC), especially among people with CVD. However, higher FGCRS was not significantly related to faster decline in PEF.

Several studies have shown that low physical and social activities are related to faster decline in PF [14, 18]. Therefore, assessing the role of physical and social activity in the association of cardiovascular burden with PF decline is needed for the primary prevention of pulmonary dysfunction. However, only two studies have explored the joint effects of physical and social activities with individual cardiovascular factors on PF decline. A cohort study showed that moderate-high levels of regular physical activities are related to a reduction in smoking-related PF decline [14]. Another longitudinal study demonstrated that active social activity might contribute to less age-related decline in PF [18]. Furthermore, FGCRS was used to assess an individual's risk of developing CVD, while specific investigations on the association of FGCRS with PF decline among CVD populations are scarce. In the current study, we found that higher FGCRS-assessed cardiovascular risk burden was related to faster decline in PF, especially among people with CVD. In addition, high levels of physical and social activities significantly mitigate the PF decline related to higher FGCRS. Our findings suggest that engagement in physical and social activities could be encouraged as a prevention strategy to delay PF decline in elderly people. We failed to find a significant interaction between FGCRS and smoking on PF. There are several explanations. First, in this study, smoking status was used as a dichotomized variable, instead of precise assessment such as cumulative tobacco consumption. Second, the sample size is limited for the examination of interactions between FGCRS and smoking on PF decline. Because the MAP study participants were healthier than the general elderly population and the prevalence of smoking was lower than in other populations. Finally, smoking is associated with elevated mortality, thus those who had heavy smoking could not be survived till old age, as a result, those who were included in the study could have less or light smoking. Thus, further large cohort studies are needed to elucidate the role of smoking in the association between FGCRS and PF.

Several mechanisms may underlie the association of cardiovascular risk burden with PF decline. First, several cardiovascular risk factors (increasing age, insulin resistance, etc.) may contribute to decreased static elastic retraction of the lung or fat deposition between the muscles and ribs, leading to the decrease of chest wall compliance and respiratory muscle strength [19, 20], thereby PF decreasing. Second, smoking, hypertension, and dyslipidemia may induce systemic inflammation and accumulation of inflammatory cells into the airways, resulting in airway structure remodeling and the destruction of the lung parenchyma, which in turn leads to a decreased PF [21, 22]. Third, regular physical activity may suppress the production of inflammatory factors, reduce airway inflammatory damage and lung parenchymal destruction, and delay the decline in lung function induced by cardiovascular risk factors through inflammation [14, 22, 23]. In addition, a high level of physical activity may reduce sedentary-induced obesity, thereby avoiding pulmonary mechanical damage caused by additional loading of the rib cage by adipose tissue, which delays the decline in lung function induced by cardiovascular factors [24–26]. Finally, active social activities may directly improve overall health by increasing positive emotions, and therefore slow down the decline in PF [27, 28].

There are several strengths in this study. Firstly, our study is a population-based cohort study with a larger sample size and longer follow-up examination, which enables to capture trajectories of PF annually. In addition, this study assessed the comprehensive cardiovascular risk burden and an aggregated indicator of PF. However, some limitations also exist. Firstly, the participants in this study were volunteers who were more well-educated than the general population, which might contribute to limitations in the generalizability of the results. Secondly, the information on CVD and depression were according to self-report, which may lead to misclassification. Thirdly, even if FGCRS was assessed at baseline, reverse causal may exist in the relationship of PF with FGCRS. However, we repeated the analysis by excluding subjects with COPD at baseline, and the correlation remained significant. Finally, potential confounding due to residual confounders (such as dietary factors [29], working environment [30], and air pollutants [31]) could not be entirely ruled out.

Conclusively, this study proves that higher FGCRS is related to a faster PF decline, especially among people with CVD. There were significant interactions between higher FGCRS and physical/social activity, and adequate physical and social activities may mitigate PF decline related to a higher cardiovascular risk burden.

## MATERIALS AND METHODS

### Study population

The MAP is an ongoing prospective cohort study of common chronic conditions in older adults [32]. Briefly, older adults without known dementia were recruited from retirement communities, senior and subsidized housing, church groups, and social service agencies in northeastern Illinois, Chicago, USA (https://www.radc.rush.edu/) [33].

**Figure 2:**
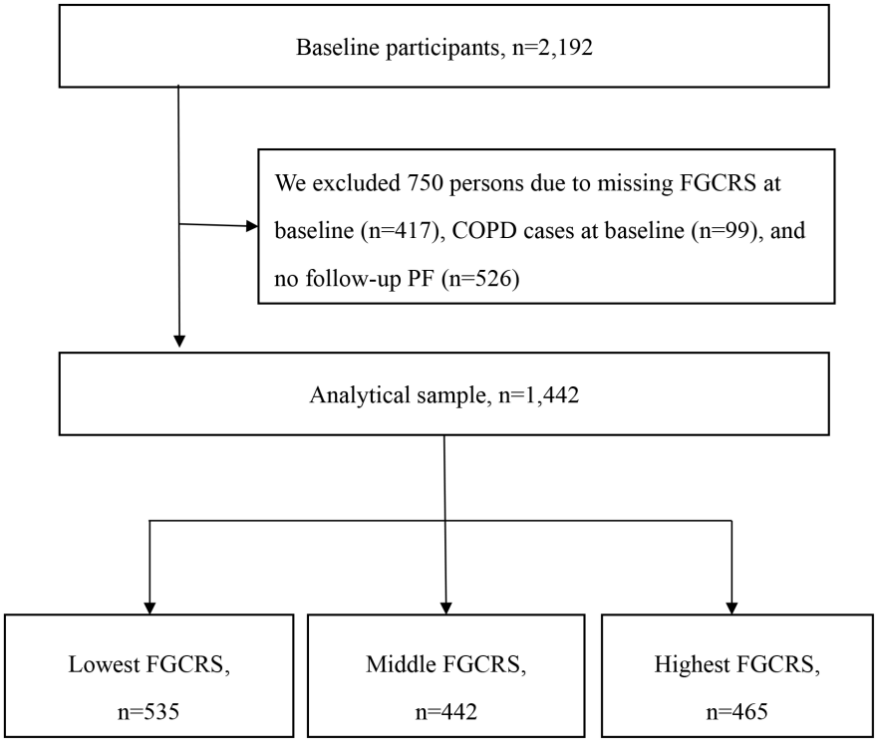
**Flow chart of the study population.** Abbreviation: FGCRS, Framingham General Cardiovascular Risk Score; COPD, chronic obstructive pulmonary disease; PF, pulmonary function.

From 1997, 2,192 participants were enrolled and annually followed for up to 22 years till 2020 [17]. Among them, 750 participants with missing baseline FGCRS (n=417), chronic obstructive pulmonary disease (COPD) cases (n=99), or lacking follow-up PF information (n=526) were excluded, and 1,442 individuals were enrolled in the current analysis (Figure 2).

MAP was approved by an Institutional Review Board of Rush University Medical Center. Written informed consent was obtained from all participants as well as a repository consent to allow their data to be shared.

### Data collection

A comprehensive clinical assessment was conducted at baseline, and information on demographics, lifestyle factors, and the histories of diseases was collected [32]. Education was represented as the years of formal school. Body mass index (BMI) was calculated by weight (kg) /height (m^2^). Smoking status was classified as never, former, or current smoker. Alcohol consumption was measured by the average amount of alcohol (grams) consumed per day over the previous year [34].

Physical activity was collected by the total weekly participation hours in walking, garden work, calisthenics, riding, and swimming [35, 36]. Social activity was rated to involve common types of social activities during the previous year. These item scores yielded the composite measurement, with higher scores reflecting more engagement in social activities [37, 38]. Both physical activity and social activity were classified as low vs. high levels by the median.

Blood pressure in the left arm was measured with a regularly tested mercury sphygmomanometer after a 5-minute interval following a standard protocol while participants were seated in a quiet room [39]. The average of the two readings was used. Hypertension was ascertained based on SBP ≥140 mm Hg, DBP ≥ 90 mm Hg, or usage of antihypertensive medication [40]. Diabetes was ascertained by HbA1c ≥ 6.5%, fasting plasma glucose ≥ 126 mg/dl, random blood glucose ≥ 200 mg/dl, diabetes diagnosis, or usage of anti-diabetic drugs [41]. Depression was identified by the doctor or the usage of antidepressants. CVD includes stroke, congestive heart failure, and heart diseases.

### Assessment of FGCRS

Baseline FGCRS was calculated by age, sex, smoking, total cholesterol, high-density lipoprotein cholesterol (HDL-C), SBP, anti-hypertension drugs, and diabetes based on the Framingham prediction model (Supplementary Tables 1, 2) [12]. FGCRS is calculated by adding the scores for all these risk factors and further dividing them into tertiles (i.e., lowest, middle, and highest). A higher FGCRS score indicates a greater risk of developing cardiovascular disease.

### Assessment of PF

PF, including FVC, FEV1, and PEF, was measured twice by a hand-held spirometer (MicroPlus Spirometer MS03, MicroMedical LTC. Kent, UK) [42, 43]. The average of the two measures was used for each subject. Raw scores of the averaged measurements (i.e., FVC, FEV1, and PEF) were transformed into z-scores. Moreover, a composite PF score was calculated by averaging the z-scores of FVC, FEV1, and PEF, respectively as previously reported [17]. Possible COPD was ascertained by FEV1 / FVC≤ 0.7 [17].

### Statistical analysis

Differences in characteristics among FGCRS categories of the study population were compared by one-way analysis of variance or Wilcoxon rank-sum tests for continuous variables, and chi-square tests for categorical variables.

The associations of FGCRS (as continuous and categorical variables) with changes in PF including PEF, FEV1, and FVC were analyzed using linear mixed-effects models and the β-coefficients and 95% confidence intervals (CIs) were estimated. The fixed effect included FGCRS, time (year), and their interaction, as well as all covariates. The random effect included random intercept and slope, allowing the individual differences at baseline and across time. Age, sex, education, BMI, alcohol consumption, physical activity, social activity, depression, stroke, congestive heart failure, and heart diseases were considered as confounders. The combined effect of two factors was assessed by creating dummy variables based on joint exposures of FGCRS and CVD, physical activity, social activity, and smoking status. Statistical interaction was tested by including the physical/social/CVD/ smoking status, FGCRS, time, and their cross-product index in the model.

In sensitivity analysis, we performed the analyses by excluding PF observations at baseline and within the first 2 years during the follow-up. *P*-values< 0.05 were considered statistically significant. All statistical analyses were performed using Stata SE 16.0 for Windows (StataCorp, College Station, TX, USA).

## Supplementary Material

Supplementary Tables

## References

[1] Thomas ET, Guppy M, Straus SE, Bell KJ, Glasziou P. Rate of normal lung function decline in ageing adults: a systematic review of prospective cohort studies. BMJ Open. 2019; 9:e028150. 10.1136/bmjopen-2018-028150 31248928 PMC6597635

[2] Brusasco V, Warner DO, Beck KC, Rodarte JR, Rehder K. Partitioning of pulmonary resistance in dogs: effect of tidal volume and frequency. J Appl Physiol (1985). 1989; 66:1190–6. 10.1152/jappl.1989.66.3.1190 2708244

[3] Baughman P, Marott JL, Lange P, Martin CJ, Shankar A, Petsonk EL, Hnizdo E. Combined effect of lung function level and decline increases morbidity and mortality risks. Eur J Epidemiol. 2012; 27:933–43. 10.1007/s10654-012-9750-2 23238697

[4] Ashley F, Kannel WB, Sorlie PD, Masson R. Pulmonary function: relation to aging, cigarette habit, and mortality. Ann Intern Med. 1975; 82:739–45. 10.7326/0003-4819-82-6-739 1094879

[5] Washko GR, Colangelo LA, Estépar RS, Ash SY, Bhatt SP, Okajima Y, Liu K, Jacobs DR Jr, Iribarren C, Thyagarajan B, Lewis CE, Kumar R, Han MK, et al. Adult Life-Course Trajectories of Lung Function and the Development of Emphysema: The CARDIA Lung Study. Am J Med. 2020; 133:222–30.e11. 10.1016/j.amjmed.2019.06.049 31369720 PMC6980254

[6] Byerley DM, Weitz CA, Richards F. Smoking and pulmonary function in five Solomon Island populations. Am J Phys Anthropol. 1992; 89:11–7. 10.1002/ajpa.1330890103 1530058

[7] Miele CH, Grigsby MR, Siddharthan T, Gilman RH, Miranda JJ, Bernabe-Ortiz A, Wise RA, Checkley W, and CRONICAS Cohort Study Group. Environmental exposures and systemic hypertension are risk factors for decline in lung function. Thorax. 2018; 73:1120–7. 10.1136/thoraxjnl-2017-210477 30061168 PMC7289445

[8] Leone N, Courbon D, Thomas F, Bean K, Jégo B, Leynaert B, Guize L, Zureik M. Lung function impairment and metabolic syndrome: the critical role of abdominal obesity. Am J Respir Crit Care Med. 2009; 179:509–16. 10.1164/rccm.200807-1195OC 19136371

[9] Davis TM, Knuiman M, Kendall P, Vu H, Davis WA. Reduced pulmonary function and its associations in type 2 diabetes: the Fremantle Diabetes Study. Diabetes Res Clin Pract. 2000; 50:153–9. 10.1016/s0168-8227(00)00166-2 10960726

[10] Allinson JP, Hardy R, Donaldson GC, Shaheen SO, Kuh D, Wedzicha JA. Combined Impact of Smoking and Early-Life Exposures on Adult Lung Function Trajectories. Am J Respir Crit Care Med. 2017; 196:1021–30. 10.1164/rccm.201703-0506OC 28530117 PMC5649988

[11] Koo HK, Kim DK, Chung HS, Lee CH. Association between metabolic syndrome and rate of lung function decline: a longitudinal analysis. Int J Tuberc Lung Dis. 2013; 17:1507–14. 10.5588/ijtld.12.0906 24125459

[12] D’Agostino RB Sr, Vasan RS, Pencina MJ, Wolf PA, Cobain M, Massaro JM, Kannel WB. General cardiovascular risk profile for use in primary care: the Framingham Heart Study. Circulation. 2008; 117:743–53. 10.1161/CIRCULATIONAHA.107.699579 18212285

[13] Pelkonen M, Notkola IL, Lakka T, Tukiainen HO, Kivinen P, Nissinen A. Delaying decline in pulmonary function with physical activity: a 25-year follow-up. Am J Respir Crit Care Med. 2003; 168:494–9. 10.1164/rccm.200208-954OC 12791579

[14] Garcia-Aymerich J, Lange P, Benet M, Schnohr P, Antó JM. Regular physical activity modifies smoking-related lung function decline and reduces risk of chronic obstructive pulmonary disease: a population-based cohort study. Am J Respir Crit Care Med. 2007; 175:458–63. 10.1164/rccm.200607-896OC 17158282

[15] Cheng ST, Leung EM, Chan TW. Physical and social activities mediate the associations between social network types and ventilatory function in Chinese older adults. Health Psychol. 2014; 33:524–34. 10.1037/hea0000026 24884906

[16] Cook NR, Evans DA, Scherr PA, Speizer FE, Vedal S, Branch LG, Huntley JC, Hennekens CH, Taylor JO. Peak expiratory flow rate in an elderly population. Am J Epidemiol. 1989; 130:66–78. 10.1093/oxfordjournals.aje.a115324 2787111

[17] Buchman AS, Boyle PA, Wilson RS, Gu L, Bienias JL, Bennett DA. Pulmonary function, muscle strength and mortality in old age. Mech Ageing Dev. 2008; 129:625–31. 10.1016/j.mad.2008.07.003 18755207 PMC2677981

[18] Crittenden CN, Murphy ML, Cohen S. Social integration and age-related decline in lung function. Health Psychol. 2018; 37:472–80. 10.1037/hea0000592 29620377 PMC5920732

[19] Ren WY, Li L, Zhao RY, Zhu L. Age-associated changes in pulmonary function: a comparison of pulmonary function parameters in healthy young adults and the elderly living in Shanghai. Chin Med J (Engl). 2012; 125:3064–8. 22932182

[20] Mannino DM, Thorn D, Swensen A, Holguin F. Prevalence and outcomes of diabetes, hypertension and cardiovascular disease in COPD. Eur Respir J. 2008; 32:962–9. 10.1183/09031936.00012408 18579551

[21] Wang Y, Xu J, Meng Y, Adcock IM, Yao X. Role of inflammatory cells in airway remodeling in COPD. Int J Chron Obstruct Pulmon Dis. 2018; 13:3341–8. 10.2147/COPD.S176122 30349237 PMC6190811

[22] van Huisstede A, Cabezas MC, Birnie E, van de Geijn GJ, Rudolphus A, Mannaerts G, Njo TL, Hiemstra PS, Braunstahl GJ. Systemic inflammation and lung function impairment in morbidly obese subjects with the metabolic syndrome. J Obes. 2013; 2013:131349. 10.1155/2013/131349 23509614 PMC3595660

[23] Das UN. Anti-inflammatory nature of exercise. Nutrition. 2004; 20:323–6. 10.1016/j.nut.2003.11.017 14990277

[24] Ferreira MS, Mendes RT, de Lima Marson FA, Zambon MP, Paschoal IA, Toro AA, Severino SD, de Oliveira Ribeiro MÂ, Ribeiro JD. The relationship between physical functional capacity and lung function in obese children and adolescents. BMC Pulm Med. 2014; 14:199. 10.1186/1471-2466-14-199 25495914 PMC4280742

[25] Gontijo PL, Lima TP, Costa TR, Reis EP, Cardoso FP, Cavalcanti Neto FF. Correlation of spirometry with the six-minute walk test in eutrophic and obese individuals. Rev Assoc Med Bras (1992). 2011; 57:380–86. 21876918

[26] Salome CM, King GG, Berend N. Physiology of obesity and effects on lung function. J Appl Physiol (1985). 2010; 108:206–11. 10.1152/japplphysiol.00694.2009 19875713

[27] Berkman LF, Glass T, Brissette I, Seeman TE. From social integration to health: Durkheim in the new millennium. Soc Sci Med. 2000; 51:843–57. 10.1016/s0277-9536(00)00065-4 10972429

[28] Crittenden CN, Pressman SD, Cohen S, Janicki-Deverts D, Smith BW, Seeman TE. Social integration and pulmonary function in the elderly. Health Psychol. 2014; 33:535–43. 10.1037/hea0000029 24884907 PMC4069253

[29] Khanam UA, Rennie DC, Davis K, Lawson JA. Are Dietary Factors Associated with Lung Function in Canadian Adults? Can J Diet Pract Res. 2020; 81:28–36. 10.3148/cjdpr-2019-023 31512487

[30] Dement JM, Welch LS, Ringen K, Cranford K, Quinn P. Longitudinal decline in lung function among older construction workers. Occup Environ Med. 2017; 74:701–8. 10.1136/oemed-2016-104205 28515054

[31] Adam M, Schikowski T, Carsin AE, Cai Y, Jacquemin B, Sanchez M, Vierkötter A, Marcon A, Keidel D, Sugiri D, Al Kanani Z, Nadif R, Siroux V, et al. Adult lung function and long-term air pollution exposure. ESCAPE: a multicentre cohort study and meta-analysis. Eur Respir J. 2015; 45:38–50. 10.1183/09031936.00130014 25193994 PMC4318659

[32] Bennett DA, Schneider JA, Buchman AS, Barnes LL, Boyle PA, Wilson RS. Overview and findings from the rush Memory and Aging Project. Curr Alzheimer Res. 2012; 9:646–63. 10.2174/156720512801322663 22471867 PMC3439198

[33] Bennett DA, Buchman AS, Boyle PA, Barnes LL, Wilson RS, Schneider JA. Religious Orders Study and Rush Memory and Aging Project. J Alzheimers Dis. 2018; 64:S161–89. 10.3233/JAD-179939 29865057 PMC6380522

[34] Wang J, Song R, Dove A, Qi X, Ma J, Laukka EJ, Bennett DA, Xu W. Pulmonary function is associated with cognitive decline and structural brain differences. Alzheimers Dement. 2022; 18:1335–44. 10.1002/alz.12479 34590419 PMC10085529

[35] Buchman AS, Boyle PA, Wilson RS, Bienias JL, Bennett DA. Physical activity and motor decline in older persons. Muscle Nerve. 2007; 35:354–62. 10.1002/mus.20702 17143881

[36] Wilson RS, Mendes De Leon CF, Barnes LL, Schneider JA, Bienias JL, Evans DA, Bennett DA. Participation in cognitively stimulating activities and risk of incident Alzheimer disease. JAMA. 2002; 287:742–8. 10.1001/jama.287.6.742 11851541

[37] James BD, Boyle PA, Buchman AS, Bennett DA. Relation of late-life social activity with incident disability among community-dwelling older adults. J Gerontol A Biol Sci Med Sci. 2011; 66:467–73. 10.1093/gerona/glq231 21300745 PMC3055280

[38] Buchman AS, Boyle PA, Wilson RS, Fleischman DA, Leurgans S, Bennett DA. Association between late-life social activity and motor decline in older adults. Arch Intern Med. 2009; 169:1139–46. 10.1001/archinternmed.2009.135 19546415 PMC2775502

[39] The hypertension detection and follow-up program: Hypertension detection and follow-up program cooperative group. Prev Med. 1976; 5:207–15. 10.1016/0091-7435(76)90039-6 935073

[40] WHO. Guideline for the pharmacological treatment of hypertension in adults. 2021. 34495610

[41] American Diabetes Association. 2. Classification and Diagnosis of Diabetes: Standards of Medical Care in Diabetes-2018. Diabetes Care. 2018; 41:S13–27. 10.2337/dc18-S002 29222373

[42] Buchman AS, Wilson RS, Boyle PA, Tang Y, Fleischman DA, Bennett DA. Physical activity and leg strength predict decline in mobility performance in older persons. J Am Geriatr Soc. 2007; 55:1618–23. 10.1111/j.1532-5415.2007.01359.x 17697103

[43] Ranu H, Wilde M, Madden B. Pulmonary function tests. Ulster Med J. 2011; 80:84–90. 22347750 PMC3229853

