# Supplementary material for: Influence of cardiovascular risk burden on pulmonary function trajectory: role of physical and social activities
Source: Aging (Albany NY). 2022 Aug 4;14(15):6081–93. doi: 10.18632/aging.204201 (PMC9417241; doi:10.18632/aging.204201)
Supplement: Supplementary Tables [file aging-14-204201-s001.pdf]

SUPPLEMENTARY TABLES

Supplementary Table 1. Framingham general cardiovascular risk score (FGCRS) calculating for women.

Points	Age (years)	HDL-C (mg/dl)	TC (mg/dl)	SBP not treated (mm Hg)	SBP treated (mm Hg)	Smoker	Diabetic	
-3				<120				
-2		60+						
-1		50-59			<120			
0	30-34	45-49	<160	120-129		No	No	
1		35-44	160-199	130-139				
2	35-39	<35		140-149	120-129			
3			200-239		130-139	Yes		
4	40-44		240-279	150-159			Yes	
5	45-49		280+	160+	140-149			
6					150-159			
7	50-54				160+			
8	55-59							
9	60-64							
10	65-69							
11	70-74							
12	75+							
Points allotted								Total

Abbreviations: HDL-C, High-density lipoprotein cholesterol, SBP, Systolic blood pressure, TC, Total cholesterol.

Supplementary Table 2. Framingham general cardiovascular risk score (FGCRS) calculating for men.

Points	Age (years)	HDL-C (mg/dl)	TC (mg/dl)	SBP not treated (mm Hg)	SBP treated (mm Hg)	Smoker	Diabetic	
-2		60+		<120				
-1		50-59						
0	30-34	45-49	<160	120-129	<120	No	No	
1		35-44	160-199	130-139				
2	35-39	<35	200-239	140-159	120-129			
3			240-279	160+	130-139		Yes	
4			280+		140-159	Yes		
5	40-44				160+			
6	45-49							
7								
8	50-54							
9								
10	55-59							
11	60-64							
12	65-69							
13								
14	70-74							
15	75+							
Points allotted								Total

Abbreviations: HDL-C, High-density lipoprotein cholesterol, SBP, Systolic blood pressure, TC, Total cholesterol.

Supplementary Table 3. Association of the covariates with the changes of pulmonary function.

Variables	Pulmonary function	FEV1	FVC	PEF
	β (95% CI)*	β (95% CI)*	β (95% CI)*	β (95% CI)*
Age	-0.49 (-0.57, -0.42)†	-0.54 (-0.63, -0.46)†	-0.52 (-0.60, -0.44)†	-0.50 (-0.56, -0.40)†
Sex	1.15 (1.07, 1.24)†	1.20 (1.10, 1.30)†	1.22 (1.12, 1.31)†	1.15 (1.05, 1.24)†
Education	0.02 (0.01, 0.03)†	0.02 (0.00, 0.03)†	0.02 (0.01, 0.03)†	0.03 (0.02, 0.04)†
Body mass index	-0.01 (-0.01, 0.00)	-0.01 (-0.02, 0.00)	-0.01 (-0.02, 0.00)	0.00 (-0.01, 0.01)
Alcohol consumption	0.00 (-0.01, 0.01)	0.00 (-0.01,0.01)	0.00 (-0.01,0.01)	0.00 (-0.00, 0.00)
Physical activity	0.17 (0.10, 0.24)†	0.19 (0.11, 0.27)†	0.19 (0.11, 0.27)†	0.15 (0.07, 0.23)†
Social activity	0.10 (0.03, 0.17)†	0.10 (0.02, 0.19)†	0.07 (-0.01, 0.15)	0.14 (0.06, 0.22)†
Depression	-0.06 (-0.15, 0.03)	-0.06 (-0.16, 0.05)	-0.02 (-0.13, 0.08)	-0.11 (-0.21, -0.01)†
Stroke	-0.10 (-0.23, 0.03)	-0.09 (-0.23, 0.06)	-0.10 (-0.24, 0.04)	-0.13 (-0.27, 0.01)
Congestive heart failure	-0.33 (-0.50, -0.15)†	-0.40 (-0.59, -0.20)†	-0.38 (-0.58, -0.19)†	-0.22 (-0.42, -0.03)†
Heart diseases	-0.08 (-0.20, 0.04)	-0.10 (-0.24, 0.04)	-0.04 (-0.18, 0.10)	-0.11 (-0.25, 0.03)

*Model adjusted for sex, age, education, body mass index, alcohol consumption, physical activity, social activity, depression, stroke, congestive heart failure, and heart diseases.

P < 0.05.

Abbreviations: FVC, Forced vital capacity; FEV1, Forced expiratory volume in one second; PEF, Peak expiratory flow.

Supplementary Table 4. Joint effect of high Framingham general cardiovascular risk score (FGCRS) and smoking status in relation to the decline on pulmonary function.

Joint exposure		No. of subjects	Pulmonary function[a]
			β (95% CI)
**FGCRS**	**Smoking status**		
Low	Non-smokers	308	Reference
Low	Smokers[b]	227	-0.009 (-0.022 to 0.004)
High	Non-smokers	550	-0.016[c] (-0.027 to -0.006)
High	Smokers[b]	357	-0.015[c] (-0.026 to -0.004)

[a]Model adjusted for sex, age, education, body mass index, alcohol consumption, depression, physical activity, social activity, and CVD.

P-interaction > 0.05.

[b]Smokers including former and current smokers.

Abbreviation: CI, confidence interval.

[c]*P* < 0.05.

Supplementary Table 5. Association of the Framingham general cardiovascular risk score (FGCRS) with the changes of pulmonary function after excluding pulmonary function at baseline and within first 2 year during the follow-up (N=1,007).

FGCRS	Pulmonary function	FEV1	FVC	PEF
	β (95% CI)[a]	β (95% CI)[a]	β (95% CI)[a]	β (95% CI)[a]
Baseline				
Continuous FGCRS	-0.018[b] (-0.030 to -0.006)	-0.019[b] (-0.035 to -0.003)	-0.018[b] (-0.017 to -0.002)	-0.022[b] (-0.039 to -0.005)
Categories FGCRS				
Lowest	Reference	Reference	Reference	Reference
Middle	-0.014 (-0.131 to 0.102)	-0.003 (-0.137 to 0.132)	-0.007 (-0.143 to 0.130)	-0.014 (-0.159 to 0.131)
Highest	-0.081 (-0.204 to 0.041)	-0.072 (-0.213 to 0.068)	-0.070 (-0.212 to 0.073)	-0.132 (-0.282 to 0.018)
Longitudinal				
Continuous FGCRS × time	-0.001[b] (-0.002 to -0.000)	-0.001[b] (-0.003 to -0.000)	-0.001[b] (-0.003 to -0.000)	-0.001 (-0.003 to 0.000)
Categories FGCRS × time				
Lowest	Reference	Reference	Reference	Reference
Middle	-0.011[b] (-0.020 to -0.002)	-0.010 (-0.021 to 0.000)	-0.011 (-0.023 to 0.001)	-0.014 (-0.031 to 0.004)
Highest	-0.013[b] (-0.023 to -0.004)	-0.015[b] (-0.025 to -0.004)	-0.013[b] (-0.025 to -0.001)	-0.010 (-0.028 to 0.007)

[a]Model adjusted for sex, age, education, body mass index, alcohol consumption, physical activity, social activity, depression, stroke, congestive heart failure, and heart diseases.
[b]$P < 0.05$.
